# The common use of improper control diets in diet-induced metabolic disease research confounds data interpretation: the fiber factor

**DOI:** 10.1186/s12986-018-0243-5

**Published:** 2018-01-15

**Authors:** Michael A. Pellizzon, Matthew R. Ricci

**Affiliations:** Research Diets, Inc, 20 Jules Lane, New Brunswick, NJ 08901 USA

**Keywords:** Diet, Purified ingredients, Fiber, Microbiota, Metabolic disease

## Abstract

Diets used to induce metabolic disease are generally high in fat and refined carbohydrates and importantly, are usually made with refined, purified ingredients. However, researchers will often use a low fat grain-based (GB) diet containing unrefined ingredients as the control diet. Such a comparison between two completely different diet types makes it impossible to draw conclusions regarding the phenotypic differences driven by diet. While many compositional differences can account for this, one major difference that could have the greatest impact between GB and purified diets is the fiber content, both in terms of the level and composition. We will review recent data showing how fiber differences between GB diets and purified diets can significantly influence gut health and microbiota, which itself can affect metabolic disease development. Researchers need to consider the control diet carefully in order to make the best use of precious experimental resources.

## Perspective

Animal models have been and continue to be crucial in understanding the etiology of metabolic disease in humans. One reason is that similar to humans, metabolic disease in lab animals can be induced by diet. Unlike human clinical work, researchers have the opportunity to carefully and easily control the animal’s environment which should include the diet. Unfortunately, there are a substantial number of studies in which improperly matched experimental and control diets are used. Such mismatched diets hamper the investigator’s ability to draw useful conclusions from what are otherwise well-designed, hypothesis-driven studies. One recurring example is the comparison of a defined, purified ingredient diet to something often referred to as ‘normal chow’ or ‘standard laboratory chow’, which typically refers to a grain-based (GB) diet (see Table 1). The term ‘normal chow’ is about as useful a description for a diet as ‘normal mouse’ would be for an animal. In addition, ‘normal chow’ suggests that 1) all chows have a consistent and known composition and 2) the use of chow as the control diet is always acceptable, neither of which is true.

**Table 1:** Typical sources of nutrients and non-nutrients in rodent purified ingredient diets and grain-based diets

Nutrients or Non-nutrients	Purified Ingredient Diet	Grain-Based Diet
	Typical Sources	Typical Sources
Protein	Casein	Dehulled soybean meal, ground corn and wheat, whey, alfalfa
Fat	Soybean oil, corn oil	Porcine animal fat, fish meal, meat meal
Carbohydrate	Corn starch, maltodextrin, sucrose	Dehulled soybean meal, ground corn, ground oats, wheat middlings
Fiber	Refined Cellulose (INSOLUBLE Fiber)	Ground corn or wheat, dried beet pulp, ground oats, alfalfa, wheat middlings (SOLUBLE and INSOLUBLE Fibers including cellulose, hemicellulose, lignins and pectin)
Micronutrients	Vitamin and mineral premixes	Most ingredients, extra micronutrients added
Phytoestrogens	None present in diet	Mainly soybean meal, alfalfa meal
Heavy Metals	None present in diet	Mainly from grains and meat meals

In this essay, our goal is to educate researchers that lab animal diets must be properly controlled and reported so that valid conclusions can be made. The use of improper control diets in metabolic disease research and the lack of adequate diet descriptions in publications has been discussed previously [1–6]. However, revisiting this topic is crucial and timely given the 1) substantial and widespread interest in the role of the gut microbiome in health and disease and 2) the undisputed effects of diet on gut microbe activity and populations. In order to appreciate why comparing these different diet types is not good science, let’s first define these diets.

## Complexity of GB diet ingredients can confound data interpretation

GB diets, which are often and unfortunately referred to vaguely as “normal chow”, “normal diet” or “standard diet” in most publications, are made with grain and cereal ingredients and animal by-products. These unrefined ingredients include ‘ground corn’, ‘ground wheat’, ‘ground oats’, ‘fish meal’, ‘alfalfa meal’, ‘brewers dried yeast’ and ‘animal fat preserved with BHA’. These ingredients contain multiple nutrients and non-nutrients, and their inclusion level in GB diets (i.e. the formula) is not only ‘closed’ (kept secret from the research community) as it is considered proprietary, but the formula itself may vary over time depending on changes in nutrient levels in key ingredients. In addition, vitamin and mineral premixes are added to these diets which supplement the unknown levels of micronutrients provided inherently from other ingredients, and in some cases in excess of the estimated requirement [7]. One the positive side, GB diets are inexpensive and have been used since the beginning of lab animal research. In addition, they are generally considered to maintain a healthy phenotype in the animal (though it can be argued ‘compared to what?’).

However, the ingredients used in GB diets are also their Achilles heel. The extent to which these ingredients are processed and the locations and conditions of where they are harvested can be a source of variation in the nutrients and non-nutrients (such as phytoestrogen levels) they contain [8–10]. Therefore, GB diets can vary significantly from batch-to-batch, from formulation to formulation and among different manufacturers. While variation in nutrient content alone should make a researcher reconsider the use of a GB diet, the presence and inconstancy of non-nutrients (which are generally not listed on the nutrition label) in these diets further adds to these concerns. Conceivably, this may inadvertently change the research question being asked in the study, leading to more time and money spent.

There is a growing list of non-nutrient entities in GB diets such as various phytochemicals (e.g. phytoestrogens, lignans) [11], toxic heavy metals (e.g. arsenic, lead) [12], nitrosamines [13, 14], endotoxins [15] and pesticides and pollutants [16–18]. Recently, Mesnage et al. [18] looked at 13 different GB diets from 5 continents and found several environmental contaminants including various pesticides, heavy metals, genetically modified grains, polychlorinated biphenyls, polychlorinated dibenzo-p-dioxins and dibenzofurans. Their levels in some cases greatly exceeded acceptable daily intakes and are highly variable among these diets. As it is apparent that these contaminants are not well controlled, it’s conceivable that they could by themselves or in combination alter the toxicological and metabolic phenotype of rodents. For example, it was found that feeding a GB diet significantly induced expression of aryl hydrocarbon receptors (AhRs) in intestinal cells – cells that modulate immunity and detoxification [19]. In contrast, expression was not induced by feeding a purified ingredient diet. However, the addition of a known AhR ligand (indole-3 carbinol) to a purified diet recapitulated the effect of the GB diet. While indole-3 carbinol is not present in GB diets, it is likely that other phytochemicals (i.e. phytoestrogens from soybean meal and alfalfa meal) or perhaps environmental contaminants such as polychlorinated dibenzo-p-dioxins may serve as AhR ligands [20].

In contrast to GB diets, purified ingredient diets (also called purified diets, semi-purified diets) use highly refined ingredients (casein, corn starch, sucrose, cellulose, soybean oil, etc.) each of which essentially contains one main nutrient and little to no non-nutrient chemicals. As a result, these diets are well-defined and have minimal batch-to-batch variability [21]. Indeed, it was through the use of purified ingredient diets that nutrient requirements for lab animals were first delineated. Furthermore, the formulas are ‘open’ and not kept secret from the scientific community. No diet is without flaws and a good example of a needed ‘improvement’ in purified diets is the inclusion of a source(s) of soluble fiber for gut health. Having said this, among nutritionally trained scientists, purified diets are considered a ‘cleaner’, more controlled diet choice compared to GB diets [4, 7, 21]. Knowing the inherent differences between GB diets and purified diets allows the researcher to design their diet study well and judge papers in which animals fed purified diets were compared directly to those fed GB diets.

**Fig. 1:**
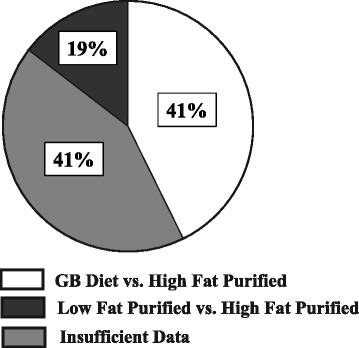
Diet comparisons in recent research publications. Pie chart showing the percentages of 69 publications evaluated (using search terms ‘mouse high fat’) that used appropriate diet comparisons (19%), that compared GB diets and purified high-fat diets (41%), and that presented insufficient information to evaluate the types of diets used (41%). The journals examined were Cell Metabolism (7 papers), Cell (1 paper), Science (1 paper), Journal of Clinical Investigation (15 papers), Nature (3 papers), Nature Medicine (4 papers), and Diabetes (the first 38 of 188 papers)

Given the inherent differences between GB and purified diets, it is clear that data produced from these diets should not be compared to each other. Yet, the incidence of improperly controlled diet studies in the lab animal literature is substantial. In 2008, Warden and Fisler [5] identified 35 papers using the search terms ‘mouse high fat’ in five high-impact journals. Of these 35 papers, only 14% used a properly matched control diet against the high-fat purified ingredient experimental diet. Forty-three percent of papers improperly used a GB diet as the control and 34% of the time, there was not enough information in the methods section to even determine what types of diets were fed. Despite this high-profile commentary, these numbers have not changed very much in recent years. We used the same search terms in the same journals, and identified 69 publications published in 2016. In 41% of the papers, data from mice fed a high-fat purified ingredient diet were improperly compared to those fed a GB diet. In another 41% of the papers, there were insufficient descriptions of the diets used and so we could not determine what the animals were fed. In only 19% of the papers was a properly matched, low-fat purified ingredient diet used in comparison to the purified ingredient high-fat diet (Fig. 1). Thus, it is possible that researchers are attributing phenotypic differences between animals fed a low fat GB diet and a high fat purified diet to differences in dietary fat, when in fact they could be due to any number of other dietary differences.

Interestingly, the authors of a recent publication [22] compared feeding a low-fat GB diet, a high-fat purified diet and a matched low-fat purified diet to C57Bl/6J mice. As they found no differences in body weight, glucose tolerance, adipokines or anxiety-like behavior between the GB or low-fat purified diet, they concluded that “chow (GB diet) may be used as an appropriate control diet in studies investigating the effects of chronic high-fat diet intake on phenotypic, metabolic and behavioral alterations”. In our opinion, this is an overreaching and misleading statement to make, given that their study, like any other, was limited in the scope of the endpoints measured. In their study, they did find differences in plasma lipids levels between the GB and low-fat purified diets, something they acknowledge could be due to differences in dietary fiber between these diet types. However, the authors fail to note that dietary fiber differences may (and based on data from the literature, *will*) affect gut morphology and the microbiome, an area of intense research. This highlights a relevant but overlooked confound that occurs any time a high fat purified diet is compared to a GB diet: the vastly different fiber types and concentrations between these diet types.

There is a growing body of evidence that the relationship between diet and metabolic disease is gut-centric. The link between the gut and weight gain was established by Turnbaugh et al. [23] who observed that lean, germ-free mice gained more adiposity after they were gavaged with cecal microbiota from obese *ob/ob* mice compared to those gavaged with microbiota from lean mice. Dramatic shifts in certain bacterial phyla can occur within a day after switching from a ‘lower fat, plant polysaccharide based diet’ (an undefined GB diet) to a high fat purified ingredient diet, and this stabilizes after only 7 days [24]. These intriguing findings suggest a rapid and powerful effect of diet on changing microbiota which precedes the development of metabolic disease. While these studies were significant contributions to our understanding of the role of gut microbiota on metabolic disease, the fact that the descriptions of the diets were limited reduces our understanding of which particular dietary factor(s) were important to the observations.

## Fiber: An X factor in GB diets that has been ignored

Of the many differences between GB and purified ingredient diets, it is arguably the level and type of dietary fiber which is most important with respect to the gut microbiome. Fiber can be generally classified as either soluble or insoluble, and there are different types of each. Bacterial fermentation of soluble fiber releases short chain fatty acids (SCFAs), which are a major supplier of energy to colonocytes and are thought to provide other benefits including prevention of diet-induced obesity, decreased adipose tissue storage, and improved insulin action [25]. An increase in SCFAs can change the gut pH, which in turn can decrease the populations of pathogenic, pH-sensitive bacteria. In contrast, insoluble fiber is generally considered to be non-fermentable and therefore does not promote gut bacterial growth and its associated effects [26]. Given these important differences in fiber, researchers working in the gut microbiota field need to educate themselves regarding the fiber level/type in the diets being fed to their animals.

What then, are the specific differences in fiber content between GB and purified ingredient diets? Crude fiber in commercial GB diets is typically reported at 5–6%, but in reality, the total fiber level in commercial GB diets varies much more and total levels are around 15–25% (~ 15–20% insoluble and 2–5% soluble, unpublished data, Pellizzon and Ricci). Fiber sources in GB diets include a mixture of cellulose, hemicellulose, lignins, and pectin, and their levels can vary significantly among different chows [27]. This is not surprising given the variable levels of fiber-containing, grain-based ingredients added to different GB diets, including ground corn, wheat, and oats, wheat middlings, soybean and alfalfa meal, dried beet pulp, and brewers dried yeast. In addition, each of these ingredients may vary in fiber level. For example, wheat middlings, a byproduct of wheat milling, can vary in fiber content (as well as other nutrients), depending on their harvest location [28]. Because of the above mentioned complexities of GB diet ingredients and their changing inclusion levels, it is impossible to know the total fiber levels and concentrations of each fiber type from diet-to-diet or even from batch-to-batch. In contrast, purified diets in general (including high- and low-fat diets) have historically contained 5% fiber, typically with refined cellulose (an insoluble fiber) as the sole fiber source. Arguments can be made that it is time to rethink the fiber component in purified diets, since it is likely that 5% cellulose is not a sufficient amount or type of fiber for optimal gut health.

In addition to the multiple differences between GB diets and purified ingredient diets already discussed, the disparity in fiber level and type alone would be expected to have important experimental consequences given their well-known effects in the intestine including adsorption of bile acids, chelation of minerals, and fermentation by bacteria in the cecum and colon. It has been known for decades that colon and cecum morphology is affected differently by GB diets and purified diets, an effect that is attributed to the differences in fiber composition of the diets [29]. Thus from a fiber perspective (in addition to others already discussed), animals fed purified ingredient diets should never be compared to those fed a GB diet. This is highlighted in a recent study by Chassaing et al. [30] and demonstrates the erroneous conclusions that can be drawn from mismatched diets. These researchers found that the colons and ceca of animals fed a high-fat purified ingredient diet were significantly smaller than those fed a low-fat GB diet. Had the GB diet been their only ‘control’, it might have been tempting to conclude that the changes in gut morphology were due to differences in dietary fat. However, when the mice were fed a low-fat *purified* diet (matched in every way to the high-fat purified diet, except in fat and carbohydrate) the mice had gut morphologies identical to those fed the high-fat diet. As there are a number of differences between these different types of diets that may have a potential influence on gut morphology, these investigators selected 2 very different factors in these diets, which were the protein type (casein vs. soy) and fiber contents. Specifically, these investigators examined if changes in protein type (casein vs. soy protein, which provided phytoestrogens), fiber levels (50 g, 100 g, and 200 g per ~ 4000 kcals), and fiber types (insoluble fiber cellulose vs. soluble fiber inulin) influenced gut morphology. Through a number of experiments, the authors showed that it was most likely the differences in dietary soluble fiber type (and not the fat level or protein type) between the GB and the purified ingredient diets that were responsible for the differences in gut size. Very recently, Dalby et al. [31] extended some of the findings of this work showing that low and high fat purified diets (10 or 60 kcal% fat and matched with the same sucrose levels and fiber as only cellulose) altered small and large intestinal microbiota composition similarly and both were very different from GB diet fed mice. While both GB diet and purified low fat fed mice had similar but significantly lower body weight, adiposity, and glucose intolerance compared to high fat fed mice, the similarities in the microbiota composition (in ileum, cecum, and colon) and cecal SCFAs of purified low and high fat fed mice suggest that other changes were responsible for the effects on the rodent metabolic phenotype. The conclusions in these studies would have been impossible to make had the GB diet been the only ‘control’. In another recent study, Desai et al. found that gnotobiotic mice with a synthetic human gut microbiota fed a purified low fat diet with very low fiber as cellulose had a significantly thinner colonic mucus layer compared to those fed a fiber-rich GB diet, something which was found to be driven by shifts in mucus-eroding microbiota and was associated with low grade inflammation and increased susceptibility to bacterial infection [32]. The differential effect of diet type on gut morphology and disease severity is also extended to mouse models of experimentally induced colitis. Miles et al. [33] observed that relative to a GB diet, feeding purified diets with either 10 or 60 kcal% fat with fiber as cellulose only increased weight loss and reduced cecum size and colon length. Again, had the GB diet been used as the only control diet, it may have been concluded that the fat level rather than diet type increased disease susceptibility. The above data suggest, not surprisingly, that changes in gut morphology and microbiota induced by diet are accompanied by changes in gut function and disease susceptibility.

Based on the above data, the differences in fiber type between a GB diet and a purified diet would be expected to influence gut morphology and metabolic health. However the extreme differences in ingredient types between GB and purified diets suggest there may be additional dietary factors influencing health. While a shift from casein to soy protein in a purified diet may not significantly influence gut morphology to the same extent as changes in dietary fiber [30], there is evidence that it can affect metabolic disease development, including body weight gain, adiposity, and plasma and liver lipids [34]. In terms of gut health, many xenobiotic compounds (including flavonoids, arsenic, and polychlorinated dibenzo-p-dioxins) in GB diets either by themselves or in combination could potentially mediate beneficial and/or toxic effects through binding gut AhR and subsequent modification of gut microbiota composition [20]. Given the complex and variable nature of GB diets as mentioned previously, it is difficult to determine the true influence of these factors on the rodent phenotype unless they are studied one at a time using a purified ingredient diet, as suggested previously [12]. Therefore, there are many potential factors in GB diets besides fiber type that can alter the rodent gut and metabolic phenotype from health to disease.

Many publications have shown the beneficial effects of adding soluble fiber to purified diets to improve health using rodent models. For example, in the context of purified ingredient diets, the addition of purified soluble fiber sources including inulin, fructooligosaccharides, and pectin can improve gut morphology [35–38] and reduce body weight and adiposity relative to insoluble cellulose [30, 38, 39] in rats and mice. In addition, other fibers including those classified as hemicellulose (which are in GB diets) such as xylans, glucans, and mannans, can improve gut morphology [40] and reduce adiposity, inflammation, and improve glucose tolerance, all of which was associated with improved mucosal barrier function [41]. Therefore, should one wish to formulate a base purified control diet that would allow rodents to have an improved gut and metabolic health profile, one easy strategy to accomplish this is to use purified soluble fiber sources. This would allow the researcher to have a better ability to control for phenotypical differences within a given study and from study to study, which would not be possible when using a GB diet, which contain a complex (and likely variable) array of fibers.

Proper experimental design needs to include an understanding of the dietary components and their potential effects on the interpretability of the data. The diet is not ‘just the food’ but a key environmental factor that can and will affect the phenotype of the animals. Given the NIH’s recent mandate to “enhance reproducibility of research findings through increased scientific rigor and transparency” in grant applications [42], researchers should be making efforts to optimize study design with respect to the diets fed. This will make the best use of funding resources, allow us to draw valid conclusions from these data and enhance our collective knowledge of these disease models. In the end, these endeavors will help us all reach our goal: the treatment and prevention of human metabolic disease.

## Abbreviations

AhR: Aryl hydrocarbon receptors
GB: Grain-based
SCFAs: Short chain fatty acids
