# Correction to: The common use of improper control diets in diet-induced metabolic disease research confounds data interpretation: the fiber factor

**DOI:** 10.1186/s12986-018-0256-0

**Published:** 2018-03-28

**Authors:** Michael A. Pellizzon, Matthew R. Ricci

**Affiliations:** Research Diets, Inc, 20 Jules Lane, New Brunswick, NJ 08901 USA

## Correction

Following publication of the original article [1] the authors noticed that the pie chart figure contains percentages (41%, 41%, 19%) that are slightly off given they don’t add to 100. They should instead read 40.6%, 40.6%, 18.8%.

## References

[1] Pellizzon MA, Ricci MR (2018). The common use of improper control diets in diet-induced metabolic disease research confounds data interpretation: the fiber factor. Nutr Metab.

